# Supplementary material for: Local Administration of Low-Dose Nerve Growth Factor Antibody Reduced Pain in a Rat Osteoarthritis Model
Source: Int J Mol Sci. 2021 Mar 4;22(5):2552. doi: 10.3390/ijms22052552 (PMC7962042; doi:10.3390/ijms22052552)
Supplement: Supplementary file 1 [file ijms-22-02552-s001.pdf]

Local Administration of Low-Dose Nerve Growth Factor Antibody Reduced Pain in a Rat Osteoarthritis Model

Yuan Tian [1], Tomohiro Onodera [1,2,*], Mohamad Alaa Terkawi [1,2], Koji Iwasaki [1], Ryosuke Hishimura [3], Dawei Liang [1], Takuji Miyazaki [1], Norimasa Iwasaki [1,2]

[1] Department of Orthopedic Surgery, Faculty of Medicine and Graduate School of Medicine, Hokkaido University, Kita-15, Nish-7, Kita-ku, Sapporo 060-8638, Japan; tyfree1001@gmail.com (Y.T.); materkawi@med.hokudai.ac.jp (M.A.T.); rockcape324@gmail.com (K.I.); 527079901ldw@gmail.com (D.L.); takuzimiyazaki@gmail.com (T.M.); niwasaki@med.hokudai.ac.jp (N.I.)
[2] Global Institution for Collaborative Research and Education (GI-CoRE), Hokkaido University, Sapporo 060-0808, Japan
[3] Department of Orthopedic Surgery, Hokkaido University Hospital, Kita14, Nishi5, Kita-Ku, Sapporo Hokkaido 060-8648, Japan; hishi_piero@yahoo.co.jp
* Correspondence: tomozou@med.hokudai.ac.jp; Tel.: +81-11-706-5935; Fax: +81-11-706-6054

Supplementary data

Table S1. Tukey's multiple comparison of weight bearing

	Groups	significant ?	Adjusted P Value
week 0	All groups	No	
week 1	MIA + Saline vs. Sham + Saline	Yes	<0.0001
	MIA + Saline vs. Sham + 100 μg	Yes	<0.0001
	MIA + 100 μg vs. Sham + Saline	Yes	<0.0001
	MIA + 100 μg vs. Sham + 100 μg	Yes	<0.0001
	MIA + 10 μg vs. Sham + Saline	Yes	<0.0001
	MIA + 10 μg vs. Sham + 100 μg	Yes	<0.0001
	MIA + 1 μg vs. Sham + Saline	Yes	<0.0001
	MIA + 1 μg vs. Sham + 100 μg	Yes	<0.0001
week 2	MIA + Saline vs. Sham + Saline	Yes	<0.0001
	MIA + Saline vs. Sham + 100 μg	Yes	<0.0001
	MIA + 100 μg vs. Sham + Saline	Yes	<0.0001
	MIA + 100 μg vs. Sham + 100 μg	Yes	<0.0001
	MIA + 10 μg vs. Sham + Saline	Yes	<0.0001
	MIA + 10 μg vs. Sham + 100 μg	Yes	<0.0001
	MIA + 1 μg vs. Sham + Saline	Yes	<0.0001
	MIA + 1 μg vs. Sham + 100 μg	Yes	<0.0001
week 3	MIA + Saline vs. MIA + 100 μg	Yes	<0.0001
	MIA + Saline vs. Sham + Saline	Yes	<0.0001
	MIA + Saline vs. Sham + 100 μg	Yes	<0.0001
	MIA + 100 μg vs. MIA + 10 μg	Yes	<0.0001
	MIA + 100 μg vs. MIA + 1 μg	Yes	<0.0001
	MIA + 10 μg vs. Sham + Saline	Yes	<0.0001
	MIA + 10 μg vs. Sham + 100 μg	Yes	<0.0001
	MIA + 1 μg vs. Sham + Saline	Yes	<0.0001
	MIA + 1 μg vs. Sham + 100 μg	Yes	<0.0001
week 4	MIA + Saline vs. MIA + 100 μg	Yes	<0.0001

	MIA + Saline vs. Sham + Saline	Yes	<0.0001
	MIA + Saline vs. Sham + 100 µg	Yes	<0.0001
	MIA + 100 µg vs. MIA + 10 µg	Yes	<0.0001
	MIA + 100 µg vs. MIA + 1 µg	Yes	<0.0001
	MIA + 10 µg vs. Sham + Saline	Yes	<0.0001
	MIA + 10 µg vs. Sham + 100 µg	Yes	<0.0001
	MIA + 1 µg vs. Sham + Saline	Yes	<0.0001
	MIA + 1 µg vs. Sham + 100 µg	Yes	<0.0001
week 5	MIA + Saline vs. MIA + 100 µg	Yes	<0.0001
	MIA + Saline vs. Sham + Saline	Yes	<0.0001
	MIA + Saline vs. Sham + 100 µg	Yes	<0.0001
	MIA + 100 µg vs. MIA + 10 µg	Yes	<0.0001
	MIA + 100 µg vs. MIA + 1 µg	Yes	<0.0001
	MIA + 10 µg vs. Sham + Saline	Yes	<0.0001
	MIA + 10 µg vs. Sham + 100 µg	Yes	<0.0001
	MIA + 1 µg vs. Sham + Saline	Yes	<0.0001
	MIA + 1 µg vs. Sham + 100 µg	Yes	<0.0001
week 6	MIA + Saline vs. MIA + 100 µg	Yes	<0.0001
	MIA + Saline vs. Sham + Saline	Yes	<0.0001
	MIA + Saline vs. Sham + 100 µg	Yes	<0.0001
	MIA + 100 µg vs. MIA + 10 µg	Yes	<0.0001
	MIA + 100 µg vs. MIA + 1 µg	Yes	<0.0001
	MIA + 10 µg vs. Sham + Saline	Yes	<0.0001
	MIA + 10 µg vs. Sham + 100 µg	Yes	<0.0001
	MIA + 1 µg vs. Sham + Saline	Yes	<0.0001
	MIA + 1 µg vs. Sham + 100 µg	Yes	<0.0001

Table S2. Tukey's multiple comparison of von frey filament test.

	Groups	significant ?	Adjusted P Value
week 0	MIA + Saline vs. MIA + 100 mg	Yes	0.0042
	MIA + Saline vs. MIA + 10 mg	Yes	0.0042
	MIA + Saline vs. MIA + 1 mg	Yes	0.0332
	MIA + Saline vs. Sham + 100 mg	Yes	0.0042
week 1	MIA + Saline vs. Sham + 100 µg	Yes	<0.0001
	MIA + 100 µg vs. Sham + Saline	Yes	<0.0001
	MIA + 100 µg vs. Sham + 100 µg	Yes	<0.0001
	MIA + 10 µg vs. Sham + Saline	Yes	<0.0001
	MIA + 10 µg vs. Sham + 100 µg	Yes	<0.0001
	MIA + 1 µg vs. Sham + Saline	Yes	<0.0001
	MIA + 1 µg vs. Sham + 100 µg	Yes	<0.0001
	MIA + Saline vs. Sham + Saline	Yes	<0.0001
week 2	MIA + Saline vs. Sham + 100 µg	Yes	<0.0001
	MIA + 100 µg vs. Sham + Saline	Yes	<0.0001
	MIA + 100 µg vs. Sham + 100 µg	Yes	<0.0001
	MIA + 10 µg vs. Sham + Saline	Yes	<0.0001

	MIA + 10 µg vs. Sham + 100 µg	Yes	<0.0001
	MIA + 1 µg vs. Sham + Saline	Yes	<0.0001
	MIA + 1 µg vs. Sham + 100 µg	Yes	<0.0001
	MIA + Saline vs. Sham + Saline	Yes	<0.0001
week 3	MIA + Saline vs. Sham + 100 µg	Yes	<0.0001
	MIA + 100 µg vs. Sham + Saline	Yes	<0.0001
	MIA + 100 µg vs. Sham + 100 µg	Yes	<0.0001
	MIA + 10 µg vs. Sham + Saline	Yes	<0.0001
	MIA + 10 µg vs. Sham + 100 µg	Yes	<0.0001
	MIA + 1 µg vs. Sham + Saline	Yes	<0.0001
	MIA + 1 µg vs. Sham + 100 µg	Yes	<0.0001
	MIA + Saline vs. MIA + 100 µg	Yes	<0.0001
week 4	MIA + Saline vs. Sham + 100 µg	Yes	<0.0001
	MIA + 100 µg vs. Sham + Saline	Yes	<0.0001
	MIA + 100 µg vs. Sham + 100 µg	Yes	<0.0001
	MIA + 10 µg vs. Sham + Saline	Yes	<0.0001
	MIA + 10 µg vs. Sham + 100 µg	Yes	<0.0001
	MIA + 1 µg vs. Sham + Saline	Yes	<0.0001
	MIA + 1 µg vs. Sham + 100 µg	Yes	<0.0001
	MIA + Saline vs. MIA + 100 µg	Yes	<0.0001
week 5	MIA + Saline vs. Sham + 100 µg	Yes	<0.0001
	MIA + 100 µg vs. Sham + Saline	Yes	<0.0001
	MIA + 100 µg vs. Sham + 100 µg	Yes	<0.0001
	MIA + 10 µg vs. Sham + Saline	Yes	<0.0001
	MIA + 10 µg vs. Sham + 100 µg	Yes	<0.0001
	MIA + 1 µg vs. Sham + Saline	Yes	<0.0001
	MIA + 1 µg vs. Sham + 100 µg	Yes	<0.0001
	MIA + Saline vs. MIA + 100 µg	Yes	<0.0001
week 6	MIA + Saline vs. Sham + 100 µg	Yes	<0.0001
	MIA + 100 µg vs. Sham + Saline	Yes	<0.0001
	MIA + 100 µg vs. Sham + 100 µg	Yes	<0.0001
	MIA + 10 µg vs. Sham + Saline	Yes	<0.0001
	MIA + 10 µg vs. Sham + 100 µg	Yes	<0.0001
	MIA + 1 µg vs. Sham + Saline	Yes	<0.0001
	MIA + 1 µg vs. Sham + 100 µg	Yes	<0.0001
	MIA + Saline vs. MIA + 100 µg	Yes	<0.0001
